# Natural Active Ingredients and TRPV1 Modulation: Focus on Key Chemical Moieties Involved in Ligand–Target Interaction

**DOI:** 10.3390/plants12020339

**Published:** 2023-01-11

**Authors:** Corina Andrei, Anca Zanfirescu, George Mihai Nițulescu, Octavian Tudorel Olaru, Simona Negreș

**Affiliations:** Faculty of Pharmacy, “Carol Davila” University of Medicine and Pharmacy, Traian Vuia 6, 020956 Bucharest, Romania

**Keywords:** TRPV1 receptors, molecular docking, mutagenesis studies, in vitro studies

## Abstract

Diseases such as cancer, neurological pathologies and chronic pain represent currently unmet needs. The existing pharmacotherapeutic options available for treating these conditions are limited by lack of efficiency and/or side effects. Transient receptor potential vanilloid 1 ion channel emerged as an attractive therapeutic target for developing new analgesic, anti-cancer and antiepileptic agents. Furthermore, various natural ingredients were shown to have affinity for this receptor. The aim of this narrative review was to summarize the diverse natural scaffolds of TRPV1 modulators based on their agonistic/antagonistic properties and to analyze the structure–activity relationships between the ligands and molecular targets based on the results of the existing molecular docking, mutagenesis and in vitro studies. We present here an exhaustive collection of TRPV1 modulators grouped by relevant chemical features: vanilloids, guaiacols, phenols, alkylbenzenes, monoterpenes, sesquiterpenoids, alkaloids, etc. The information herein is useful for understanding the key structural elements mediating the interaction with TRPV1 and how their structural variation impacts the interaction between the ligand and receptor. We hope this data will contribute to the design of novel effective and safe TRPV1 modulators, to help overcome the lack of effective therapeutic agents against pathologies with high morbidity and mortality.

## 1. Introduction

Over the years, medicinal plants have contributed immensely to pharmaceutical development. Furthermore, medicinal plant drug discovery continues to provide lead compounds effective in treating currently unmet needs, such as cancer, HIV/AIDS, Alzheimer’s and chronic pain [1]. Artemisinin, a sesquiterpene lactone isolated from *Artemisia annua* L. and used as an anti-malarial [2], galantamine, isolated from *Leucojum aestivum,* approved for the treatment of Alzheimer’s disease [3], paclitaxel, isolated from *Taxus brevifolia* [4] and vinblastine from *Catharanthus roseus*, used as antineoplastics [5] are only a few examples of the plant-developed drugs which are currently being used in therapeutics.

The advances made in molecular biology led to the identification of their specific molecular targets, offering a full view of the therapeutical utility of these natural components. Transient receptor potential vanilloid 1 (TRPV1) ion channel is such a target. Its activation regulates various biological responses, such as cell apoptosis and proliferation [6], nociception and body temperature (Tb) [7], control of metabolism and glucose homeostasis [8], and regulation of bladder function [9]. Its modulation by both natural and synthetic compounds has proven useful in the treatment of various diseases.

Preclinical studies indicated that TRPV1 modulators can be effective in the topical treatment of pruritus [10], atopic dermatitis [11], and psoriasis [12]. These results were confirmed in phase II and III clinical trials [13]. Both agonists and antagonists of TRPV1 channels demonstrated anticonvulsant effects in preclinical studies [14]. They were effective in reducing seizures in GEPR-3s rats (genetically epilepsy-prone) [15] or seizures induced by electroshock [16], pentylenetetrazol [17] or kainic acid [18]. This indicates a high-spectrum anticonvulsant effect which should be further confirmed by clinical trials. Furthermore, both agonists and antagonists seem to be effective in depression [19,20,21] and bladder hyperactivity [22,23,24,25,26]. Similar effects of agonists and antagonists result from the fact that after an initial activation of the channel, agonists induce its desensitization [27,28,29].

Multiple studies support the role of TRPV1 receptor in pain and inflammation [30,31,32,33,34], TRPV1 being one of the most important targets for the development of new analgesics [35]. TRPV1 antagonists showed an analgesic effect in animal models of inflammatory, neuropathic pain and pain associated with cancer or osteoarthritis [36,37,38,39,40,41,42]. Agonists also possess an analgesic effect by reversible desensitization of the channel [30]. Both agonist and antagonists reached clinical trials for the treatment of various types of pain: muscle and bone [43,44,45], neuropathic [30,43,44,45], dental [46,47], eye [48] and rectal pain, as well as of pain in migraine [49,50], postherpetic neuralgia [43,44,45,51], and osteoarthritis [30,52,53,54,55,56].

However, in the case of agonists, systemic administration leads to side effects of increased severity such as respiratory damage [57,58]. Thus, only topical preparations with limited effectiveness have been developed [30]. The TRPV1 agonist capsaicin is currently the only one approved for the treatment of muscle, bone, neuropathic pain and migraine, and it exists only as cream with low concentrations or as transdermal patches [30,57]. Antagonists successfully reached stage II and III clinical trials. Despite their analgesic effectiveness, most clinical trials have been suspended due to the occurrence of adverse reactions such as severe hyperthermia and impaired noxious heat sensation [59].

Structural studies have indicated that the analgesic and adverse effects are produced by different modes of activation of TRPV1. Thus, TRPV1 can be activated by capsaicin, protons (low pH) and heat, by spatially distinct mechanisms [60]. The canonical vanilloid agonist capsaicin binds to a voltage-sensing subdomain in the intracellular leaflet of the channel [61]. The proton-sensing mechanism is localized primarily on the extracellular loops of the TRPV1 pore subdomain [62]. The heat-activation mechanism is not well understood, and there are some data indicating that it depends on amino acid residues located in the transmembrane region [60].

The recently published structures of TRPV1 allow the application of combined in silico techniques, such as molecular docking and pharmacophore modeling, to better understand the protein–ligand interactions occurring at the binding site [63,64].

This narrative review focuses on identifying potent natural compounds which modulate TRPV1 channels, clearly classifying them in agonists and antagonists. Further, we summarize the major interactions between these ligands and the molecular target, highlighting the involved pharmacophores and amino acid residues. We try to distinguish pharmacophoric interactions between the protein and its ligands with both safe and unsafe profiles. This type of understanding could lead to the development of novel chemical entities with the desired therapeutical profile and less undesirable side effects.

## 2. The Structure of TRPV1 and Key Features of Its Interaction with Endogenous Ligands

Understanding the structure of TRPV1 channel, binding sites of endovanilloids and key structural features of the endogenous ligands is critical for the development of novel active substances to modulate this receptor.

TRPV1 is structurally characterized as a homotetrameric channel. Each of the four subunits contains six transmembrane domains (S1–S6) [65,66]. Each monomeric chain comprises a total of 838 amino acids, with amino acid residues 433–684 form the transmembrane domains [67,68]. The transmembrane region comprises six helices (S1–S6) forming the voltage sensor-like domain (S1–S4) and an inner pore region (S5–S6) [69]. Transmembrane domains 5 and 6 are linked by a hydrophobic loop S4S5-linker [70] and are involved in the formation of the channel pore. The ion channel pore is formed by the selectivity filter and the pore helix. Residues from the lower part of the S6 helix behave as an activation gate [71]. The different TRPV subtypes have different pore radii regulating the selectivity of the channels [66]. Binding of an activating ligand causes sequential opening of both gates, which are coupled allosterically [72]. As mentioned in the Introduction, TRPV1 has multiple binding sites inducing multiple binding modes: VBP is a hydrophobic cavity constituted by residues Tyr512, Ser513, Thr551 and Glu571 in the human TRPV1 sequence. The binding site for protons is conserved between species and it is located extracellularly in the loop between S5 and S6, with residues critical for its Glu600 and Glu648 being critical for proton activation [73]. Several structural features of TRPV1 are involved in its heat-induced activation. Despite the phenomenon is not completely understood, it seems that heat activation is based on conformational changes in the outer pore-turret [74,75]. Activation of the receptor leads to cation influx resulting in the transfer of an action potential to the neuron and subsequently to the brain. This is why agonists of TRPV1, which trigger the open state of the channel, determine burning sensations, stinging or tightness. Conversely, capsazepine blocks TRPV1 receptor, inhibiting calcium influx induced by VBP-binding agonists but not by other painful stimuli such as heat or acids.

TRPV1 also has a large cytoplasmic domain consisting of two intracellular terminal sequences [76]: the long N-terminal region formed by multiple ankyrin repeats, responsible for the activation of the channel under the action of agonist substances such as capsaicin, resiniferatoxin or high temperature (50 °C) [77], and the short C-terminal region with a role in the stability and function of the receptor [59,68,78,79]. These domains are critical scaffolds for interactions with other proteins. Furthermore, they contain binding sites for regulators of TRPV1, such as calmodulin or ATP [59,79].

TRPV1 has multiple endogenous ligands—anandamide (AEA), *N*-arachidonoyl dopamine (NADA), *N*-oleoylethanolamine (OEA), lysophosphatidic acid, oleoyldopamine (OLDA) and other molecules [80,81]. AEA, NADA, OEA and OLDA bind to VBP assuming a “tail-up, head-down” binding configuration, as does capsaicin. However, the details in their binding modes are different. A hydrogen bond is formed between NADA and OLDA, but not between AEA, OEA and residue Thr551 [82]. For AEA, the hydroxyl group in its head likely formed a hydrogen bond with residue Glu571, which is consistent with the observation that mutation of Glu571A largely abolished AEA activation [82].

Muller et al. investigated the hypothesis that AEA activates the channel by linking to the voltage-sensing-like domain formed by S1–S4, due to its flexibility and appropriate location or by binding to the capsaicin-binding pocket [83]. In contrast, unlike the hydrogen bond between the amide group of capsaicin and residue Thr551, docking suggested that there was no hydrogen bond between AEA and residue Thr551, which was supported by the results that mutation of Thr551V did not largely alter the AEA activation. Therefore, based on such observations we believe that the hydrogen bonding network between the endocannabinoids and TRPV1 channel is distinct to that of capsaicin, although these ligands bind to the same VBP with similarities in their chemical structures. Furthermore, molecular docking studies on the binding of anandamide to TRPV1 channel revealed that the ethanolamine group of anandamide interacted with the Tyr554 residue of the channel. The researchers revealed that the hydroxyl moiety of anandamide also binds to Tyr551 of the vanilloid binding site [83], but this interaction does not activate the receptor. An interaction between anandamide and Ser512 was also identified [83]. Thus, they concluded that Tyr554 and Tyr555 are the amino acids required for the ligand–receptor interaction leading to receptor activation [83].

OEA likely adopted a similar binding configuration as AEA [82]. *N*-arachidonyl-dopamine differs structurally from anandamide: it has an identical neck and tail, and their heads differ only by the presence of the benzene ring. The heads of AEA and NADA point toward Tyr512. Based on this structural similarity, Li et al. studied the interaction of this ligand with TRPV1 channel and just like in the case of anandamide, they identified residue Tyr512 as important for binding formation, with the bulky benzene ring being about 3.48 Å apart from the side chain of residue Tyr512 [82].

Another TRPV1 channel ligand is lysophosphatidic acid. To determine how this molecule binds to TRPV1, mutagenesis studies were performed by Nieto-Posadas et al. in 2012. The researchers started from the idea that lysophosphatidic acid binds to the same site as PIP2 (phosphatidylinositol 4,5-bisphosphate). In this regard, they observed that this endovanilloid interacts with the Lys710 residue located in the C-terminal region through an electrostatic mechanism, but also by involving the acyl group of the lysophosphatidic acid structure in the formation of hydrophobic bonds with this amino acid residue [84]. OLDA adopts a slightly different binding configuration, where its head bounds deeper inside the VBP.

## 3. Natural Modulators of TRPV1 Channel

### 3.1. TRPV1 Agonists

#### 3.1.1. Capsaicin and Related Compounds (Vanilloid Derivatives)

**Figure 1:**
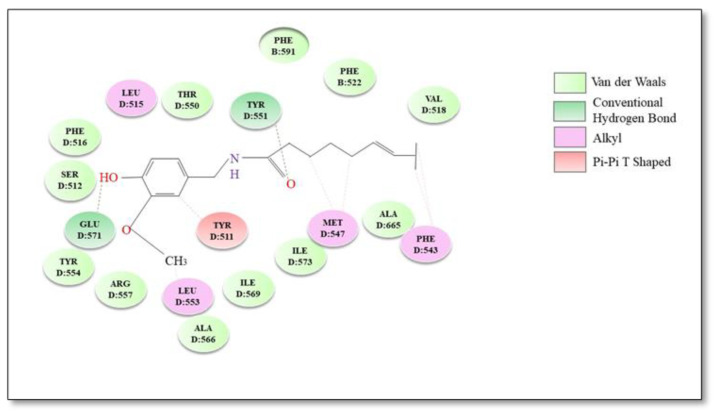
Molecular docking 2D diagram of capsaicin with TRPV1 receptor.

Capsaicin (*trans*-8-methyl-*N*-vanillyl-6-nonenamide) is a high-pungency spice-derived substance of the genus *Capsicum*, which belongs to the Solanaceae family [85]. Capsaicin binds to the intracellular side of hTRPV1. The amide oxygen of capsaicin forms a hydrogen bond with Tyr551 located in the S2–S3 intracellular loop, and the hydrophobic tail crosses into the membrane [86]. In addition to Tyr551, Ser512 is critical for capsaicin activation of TRPV1 channels. The binding pocket is located between the flexible S3 region and the voltage sensor segment. Ser512 serves as a bolt for the VBP: in the apo state it points downward, allowing capsaicin to enter VBP; upon capsaicin binding the bolt switches upward, blocking the capsaicin molecule inside [82]. The bound capsaicin is orientated “head-down tail-up” and the complex ligand/protein is stabilized by an additional hydrogen bond between the hydroxyl group in the capsaicin head and the carboxyl group of Glu571 on the S4–S5 linker [87]. Furthermore, numerous hydrophobic interactions are established between the tail of capsaicin and various amino acid residues in TRPV1 structure [87]. Two hydrophobic clusters can be distinguished: Leu515, Ile573, Phe587 and Leu669 that anchor the vanillyl ring and Met547, Ala665 and Phe543 which interact with the hydrophobic tail of capsaicin [86]. Other important residues for the interaction between capsaicin and TRPV1 are Thr550 and Tyr511 [88]. The best binding conformations and possible binding mode interactions between TRPV1 and capsaicin are illustrated in Figure 1.

Following the formation of the ligand–receptor interaction the receptor is activated, and an influx of calcium occurs, resulting in pain sensation. After the initial stimulation, capsaicin determines receptor desensitization with the installation of an analgesic effect [88,89]. The plants from the *Capsicum* genus produce a diverse array of structurally related compounds frequently named capsaicinoids. There are more than twenty capsaicinoids identified, the most important being dihydrocapsaicin, nordihydrocapsaicin, homodihydrocapsaicin and homocapsaicin. All derivatives have similar behavior towards TRPV1 channel [90]. The capsaicin and capsaicinoids are produced by all plants of the genus *Capsicum*, with the exception of *Capsicum annum* (bell pepper) [91].

Derivatives containing a vanilloid group, such as vanillylmanderic acid, vanillic acid, vanillyl alcohol and vanillyl butyl ether affect the capsaicin-binding pocket of mTRPV1 in a similar manner, however, the shortening of the carbon chain results in a decrease in sensitivity for TRPV1 [87,92].

Resiniferatoxin, isolated from *Euphorbia resinifera*, is a phorbol-related diterpene with a vanillyl moiety, used in traditional medicine for its analgesic properties [93]. It is a potent TRPV1 agonist. Its potency is substantially higher than that of capsaicin. Resiniferatoxin also binds VBP. The principal difference consists in that the vanilloid group of resiniferatoxin interacts with Tyr555 instead of Tyr511 [94]. The methoxy group at the 3-position in the vanilloid ring interacts via hydrophobic bonds with Tyr555. Other hydrophobic interactions involve the side chains around the potential resiniferatoxin binding site: Leu515, Phe543 and Asn551. Mutation of conserved residues in the pore-forming region of S6 can differentially affect capsaicin and proton sensitivity with relatively little effect on resiniferatoxin binding [94].

Pungent substances isolated from *Zingiber officinalis*, such as shogaols, gingerols, paradols and zingerone, have multiple therapeutic actions, including broad-spectrum analgesia, anti-inflammatory and anti-cancer effects [95,96,97]. They activate TRPV1 by binding the S4–S5 linker, more precisely the two residues that form a hydrogen bond with capsaicin: Thr551 and Glu571. The complex 6-Shogaol has a similar structure to capsaicin: it contains the same vanilloid head and an equal-length aliphatic tail. However, the complex 6-shoagol/TRPV1 seems to be more stable, possibly owing to the existence of the C═C bond at the base of its tail, which limits the tail’s rotational freedom [87,98]. Compared to 6-shogaol, 6-gingerol contains a hydroxyl group instead of a double bond in the tail. The additional hydroxyl group can potentially participate in the formation of a hydrogen bond, like its neighboring carbonyl group [98]. For shogaol, elongating the acyl chain length led to weakened potency, whereas the potency of gingerols was not dependent on acyl chain length [99]. Zingerone might directly interact with the channel pore when bound inside the ligand-binding pocket: it might interact with Thr671 on the S6 segment, suggesting that this tail-less ginger compound might take two alternative binding poses—a vertical pose used by capsaicin and other ginger compounds and a novel horizontal pose in which it forms a bridge between S4 and S6 [98].

**Figure 2:**
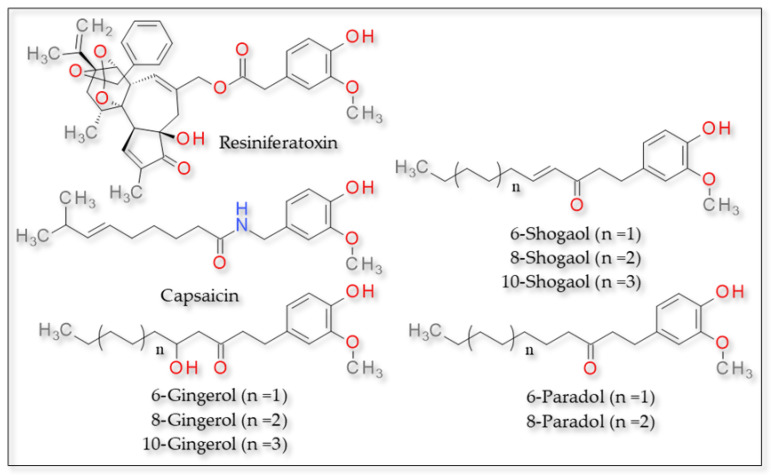
TRPV1 agonists—structure of resiniferatoxin, capsaicin and other derivatives activating TRPV1.

The compounds 2-paradol, 4-paradol and 8-paradol established with TRPV1 the same hydrogen bonds as capsaicin, involving the hydroxyl group of the derivatives and residues Leu32 and Thr28 of the receptor. However, 6-paraodol established a hydrogen bond via the hydroxyl group with residues Gln143 and Glu140 and via the keto group with Gln135, while 10-paraodol presented no hydrogen bonds. All compounds presented hydrophobic interactions between the aliphatic tail and various residues of the receptor [97]. The structures of these compounds can be found in Figure 2.

#### 3.1.2. Agonists with Various Structures

**Figure 3:**
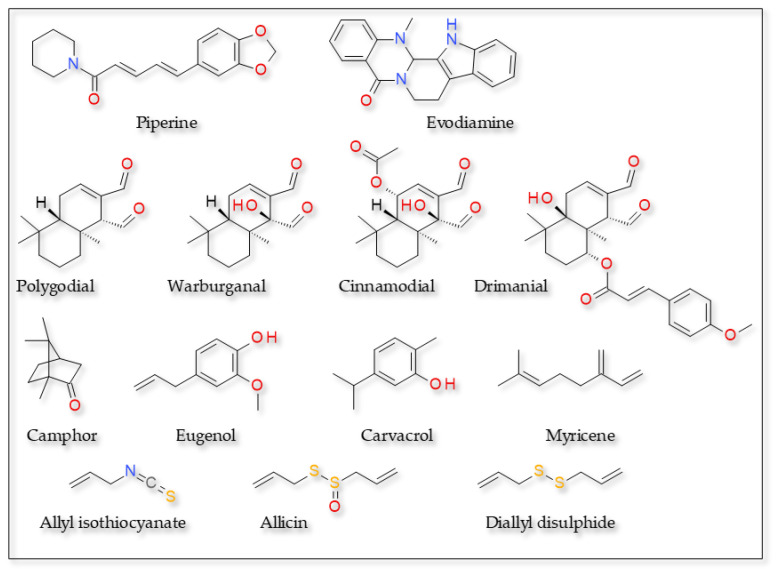
TRPV1 agonists—various structures.

Numerous TRPV1 agonists with structures different from that of capsaicin and related derivatives were isolated from medicinal and edible plants (Figure 3).

##### Alkaloids

Some alkaloids, such as rimocidin A [100], evodiamine [101], butacaine [102], piperine [103] and nicotine [104], stimulate calcium influx by activation of TRPV1 channels. Information on major ligand/target interactions exists for piperine and evodiamine.

Piperine is the major bioactive component of pepper, responsible for its pungency. This naturally occurring alkaloid has various effects and beneficial therapeutic properties, being used in traditional medicine for the treatment of pain, rheumatism, influenza, muscular pains and fever [105]. It is a weak agonist of TRPV1, binding to the same ligand-binding pocket as capsaicin. It interacts with Thr671, not with residues Thr551 and Glu571, which form hydrogen bonds with capsaicin. Extrapolation from experimental data indicated that the maximal level of channel activation induced by piperine is substantially lower than that of capsaicin (252.3 ± 38.1 µM vs. 0.1 ± 0.003 µM) [106].

Evodiamine, the major bioactive alkaloid identified in *Evodia rutaecarpa*, also activates TRPV1 [107]. Molecular docking and simulation showed that evodiamine occupies the binding pocket formed by Ser510, Tyr511, Leu515, Tyr555, Met568, Ile569, Glu570 and Lys571. The indole’s benzene ring of evodiamine established a hydrophobic interaction with Tyr511, while the benzene of the quinazoline moiety 5 formed an aromatic π-π interaction and hydrophobic interactions with Tyr555. Additionally, it made two H-bonds between the formyl carbonyl oxygen and the indole nitrogen with the side chains of Lys571 and Ile569, respectively [107].

##### Unsaturated Dialdehyde Terpenes

Cinnamodial (and its non-aldehydic derivatives cinnamosmolide and cinnamolide) are terpenes isolated from the bark of *Cinnamosma fragrans* (Cannellaceae). They induce Ca^2+^ uptake in rat DRG and inhibit a specific [3H]-RTX binding site in rat spinal cord membranes. At low concentrations, they evoke Ca^2+^ uptake in a concentration-dependent manner, and this increase is prevented by the competitive vanilloid receptor antagonist, capsazepine. Conversely, at higher concentrations these compounds caused a blockade of Ca^2+^ uptake. Thus, it should be taken into account that cinnamodial is a partial TRPV1 agonist [108]. Notably, cinnamon could upregulate the body temperature (Tb) in cold environments and activate the brown adipose tissue [109].

Polygodial and drimanial are unsaturated dialdehydes present in water pepper (*Polygonum hydropiper*) and the pepper-bark tree (*Warburgia salutaris*), used in traditional medicine for various effects: analgesic effect and in treating respiratory diseases, diabetes and cardiovascular diseases [110]. Their in vivo systemic administration produces marked antinociceptive, anti-inflammatory and antiallergic effects [111,112,113]. These effects are partly mediated by modulation of TRPV1 receptors, and these compounds inhibit specific resiniferatoxin binding in the rat spinal cord [114]. In cultured rat trigeminal neurons, polygodial and drimanial significantly increased the intracellular Ca^2+^ levels, an effect that was significantly prevented by capsazepine. However, this effect is dose-dependent: it occurs at low concentrations of ligands, while at high concentrations, Ca^2+^ uptake is blocked [115].

Other unsaturated 1,4-dialdehydes terpenes reported to act as agonists of TRPV1 include warburganal, scalaradial, aframodial, ancistrodial, merulidial and drimenol [108,114]. However, information lacks on the interactions established by this type of ligand and TRPV1.

##### Substances with Pronounced Electrophilic Character

Compounds such as allyl isothiacyanate [116], allicin [117] and diallyl disulfide [118] activate TRPV1 receptor. They are natural pungent compounds in garlic. They induce sensitization of the responses to heat, the underlying mechanism being sensitization of TRPV1 [116]. The mechanism underlying the activation of TRPV1 involves the covalent binding to Cys158 (human) or Cys157 (rat), a residue critical for the sensitivity to cysteine-modifying agents. However, for the TRPV1/allyl isothiocyanate interaction, the residue Ser513 seems to also be critical, suggesting the ligand may bind to the capsaicin binding site, despite the lack of the vanilloid motif [119]. The effect of diallyl sulfide is slower and less intense than that of allicin [120].

##### Monoterpenes

Monoterpenes such as carvacrol [121], menthol [122], umbellulone [123], isovelleral [108,114] and camphor [124] are known to activate TRPV1 channel. The best described interaction is the one between TRPV1 and the terpenes camphor and myrcene. Camphor is isolated from the wood of Cinnamomum camphora and is used in the treatment of respiratory diseases, or applied topically, as an antipruritic and analgesic [124]. It activates TRPV1 in a dose-dependent manner independent of the vanilloid-binding site and desensitizes the channel more rapidly and completely than does capsaicin. Capsaicin binding results in pore dilation, increasing the selective passage of large cations over sodium ions. Camphor acts on the channel by inducing perturbations in the outer pore region, changing the selectivity filter of the channel. It induces conformational changes in the outer pore region between transmembrane domains 5 and 6 of TRPV1 (Leu630-Phe640) [125]. Additionally, Thr633 is also essential for camphor activation. Thr633 is a specific residue located in the middle of the pore helix that is also critical for direct activation of TRPV1 by protons [126].

Eugenol functioned as a weak, partial TRPV1 agonist and a competitive capsaicin antagonist at pH 7.4. The inhibitory effect of eugenol on TRPV1 requires TRPV1 channel activation. Under low pH conditions, low concentrations of eugenol only enhanced the proton-induced TRPV1 currents. Eugenol does not affect the heat-induced TRPV1 channel activity. Differently from capsaicin, eugenol lacks both an amide group and a long aliphatic tail. Hence, TRPV1 activation required a higher concentration compared to that of capsaicin [127]. The acyclic monoterpene myrcene occurs in several essential oils, such as thyme or ylang-ylang [128]. It activates TRPV1 channel, however, the activation is not associated with the pore dilated open state that is associated with capsaicin-induced currents [129].

Furthermore, myrcene-induced TRPV1 currents are highly sensitive to internal Ca^2+^. Thus, under high internal Ca^2+^ conditions myrcene could occupy TRPV1 without activating it but only affecting its subsequent availability to other stimuli. Molecular docking data indicate myrcene interacts hydrophobically, non-covalently with Arg491 and Tyr554 residues and close to the S4–S5 linker. Various other interactions are established between carbons of myrcene and residues Phe488, Asn437, Phe434, Tyr555, Ser512, Glu513 and Phe516, residues conserved between rat and human TRPV1 [129].

##### Phytocannabinoids

Cannabis species contain various active components, with the most prevalent being Δ9-tetrahydrocannabinol, cannabidiol and cannabinol. Cannabidiol is approved for the treatment of seizures associated with Lennox-Gastaut syndrome or Dravet syndrome in pediatric patients [130] and is reported to possess analgesic, anxiolytic, and anti-inflammatory properties [131]. Cannabidiol inhibits the binding of [3*H*]-resignification at a micromolar concentration and increases intracellular free Ca^2+^ in hTRPV1-HEK293 cells, being a full agonist of TRPV1 [132]. It activates TRPV1 in the undilated state compared to capsaicin [129], the residues critical for the interaction being Tyr554 and Arg491 located in the S4–S5 linker. Cannabidiol did not influence Tb in rats following intraperitoneal administration [133].

The major interactions between TRPV1/ligand as well as the involved amino acid residues and chemical moieties are presented in Table 1.

### 3.2. Antagonists

**Figure 4:**
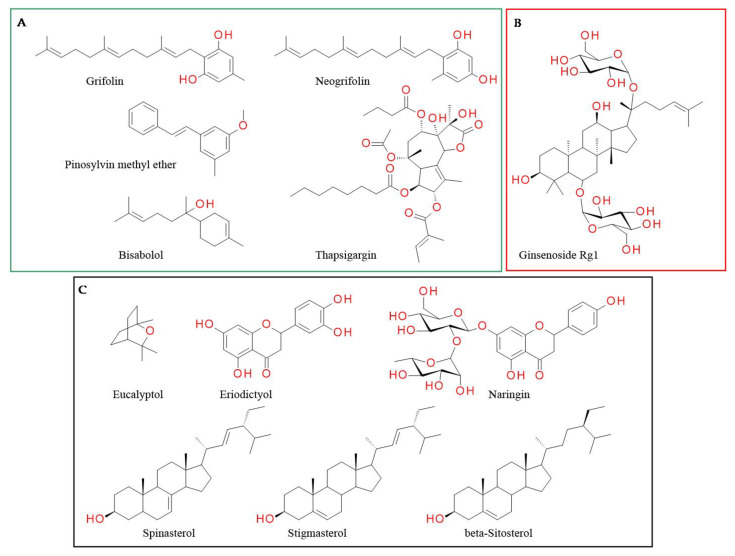
TRPV1 antagonists: (**A**) those whose effect on body temperature is not reported; (**B**) those that increase body temperature; (**C**) those not influencing body temperature.

Antagonists of the transient receptor potential vanilloid-1 (TRPV1) channel alter Tb: most cause hyperthermia, while some produce hypothermia or have no effect on Tb [139,140,141,142,143,144]. The knock-out of TRPV1 in mice or TRPV1 antagonists induce prolonged hyperthermia, an undesired side effect, upon exposure to warm ambient temperature, which led to the interruption of the phase II clinical studies involving TRPV1 antagonists [60]. Thus, we classified natural compounds based on their effect on Tb (Figure 4).

This effect was shown to depend on the effect of the antagonist on all three binding modes of TRPV1 receptor. Thus, first-generation (polymodal) TRPV1 antagonists potently block all three TRPV1 activation modes. Second-generation (mode-selective) TRPV1 antagonists potently block channel activation by capsaicin, but exert different effects (e.g., potentiation, no effect or low-potency inhibition) in the proton mode, heat mode or both. In rats, only one mode of TRPV1 activation—by protons—regulates the thermoregulatory responses to TRPV1 antagonists. When a TRPV1 antagonist blocks tonic TRPV1 activation by protons, hyperthermia occurs, while potentiation of TRPV1 activation induces hypothermia. In humans, the hyperthermic effect depends on the antagonist’s potency to block TRPV1 activation by both heat and protons [60]. Consequently, polymodal TRPV1 antagonists increase Tb [145,146,147], whereas the mode-selective (second-generation) compounds potently block the capsaicin and heat activation modes or just solely the capsaicin mode, while not affecting/potentiating the remaining modes [60].

#### 3.2.1. Compounds Whose Effect on Tb Is Not Reported

Grifolin and neogrifolin, isolated from *Peperomia galioides, Rhododendron dauricum* and *Albatrellus* sp., inhibited TRPV1 receptor in the low micromolar range. The IC50 binding values for grifolin and neogrifolin are 18.8 µM and 30.8 µM, respectively [148]. However, we found no information on the mode of binding of these triprenylphenols with the receptor or on their effect on Tb. Pretreatment with pinosylvin methyl ether (PME) injected intraplantarly (300 μM, in saline) significantly decreased the number of paw flinches induced by capsaicin. PME increased the EC50 of capsaicin without altering maximal response, indicating that PME inhibits TRPV1 through a competitive mechanism. Conversely, resveratrol, which is a stilbenoid with a similar structure, does not activate TRPV1 channel but does activate the TRPA channel [149].

Various alkaloids such as yohimbine [150], voacangine [151], pellitorine [152], monanchomycalin B [100] and pulchranins [153] inhibit capsaicin-induced Ca^2+^ influx. Yohimbine, an indole alkaloid isolated from the bark of *Pausinystalia yohimbe* Pierre, inhibits TRPV1 and the capsaicin-induced firing activity of DRGs. Doses of 1.0 and 4.0 mg/kg produced significant antinociception [154]. In preclinical studies, yohimbine showed a hypothermic effect [155], however, this effect was mainly mediated via adrenergic and serotoninergic pathways [155].

Thapsigargin, a sesquiterpene lactone isolated from *Thapsia garganica*, inhibits TRPV1-mediated [Ca^2+^]-uptake and blocks resiniferatoxin binding sites in rTRPV1-CHO cells. However, it has high toxicity owing to the affinity for a sarco/endoplasmic reticulum Ca^2+^-ATPase pump, which is critical for cell survival. Extensive research was undertaken to understand the interaction between the pump and thapsigargin, however, information on its binding to TRPV1 is still lacking. Thapsigargin-based prodrugs are currently under development as anti-cancer therapies with acceptable tolerability, as the lactone induces cancer cell apoptosis in both proliferative and quiescent phases of the cell cycle [156].

Bisabolol is a sesquiterpene which can be found in a wide variety of plants, shrubs and trees. It is the main active ingredient in the essential oil of *Matricaria chamomilla* (up to 50%) [157]. It has analgesic properties correlated with TRPV1 blockade. Bisabolol possesses high affinity for the geometric center of the TRPV1 molecule. The most strongly interacting amino acids are Ala680 and Asn687 (hydrophobic bonds) and Gly683 (hydrogen bond with 2-hydroxi moiety) [158].

#### 3.2.2. Compounds Increasing Tb

Ginsenoside Rg1, one major bioactive component of *P. notoginseng*, significantly decreased capsaicin-induced calcium influx in HaCaT and HEK 293T-TRPV1 cells, similar to capsazepine. Ginsenoside Rg1 (10 µM) significantly decreased proton-activated calcium influx in HaCaT cells by 19.76% [159]. A study investigating the use of this compound as an anti-depressant demonstrated that oral doses of 16 mg/kg prevented the reserpine-induced hypothermia [160]. Thus, this compound increases body temperature, which is in accordance with the previously presented data.

#### 3.2.3. Compounds Not Influencing Tb

Eucalyptol (1,8-cineol), the main component in the essential oil of eucalyptus leaves and other medicinal plants, is extensively used for its anti-inflammatory properties. In preclinical studies, eucalyptol inhibited acid-, formalin- and heat-evoked pain behavior [161,162]. Furthermore, its antinociceptive effect (oral administration, 200 mg/kg) was significantly inhibited by pretreatment with capsazepine (a competitive TRPV1 channel antagonist), suggesting that eucalyptol blocked TRPV1 channel, interacting with Ala680, Gly683 and Asn687 [163]. No significant change in the locomotor performance or Tb was reported [163].

Various flavonoids induced TRPV1-mediated antinociceptive effects, such as reduction in carrageenan-, capsaicin- and chronic complete Freund’s adjuvant (CFA)-induced mechanical and thermal hyperalgesia [164,165,166,167]. Flavonoids with TRPV1 antagonistic properties included various compounds: naringenin [168], cochinchinemin A and B, loureirin B [169], gomisin A [170], eriodictyol [165], quercetin [171] and vitexin [164].

Eriodictyol is a flavonoid widespread in citrus fruits, vegetables and various medicinal plants, such as *Eriodictyon californicum* and *Eupatorium arnottianum*. It has a broad spectrum of pharmacological activities, including analgesic effects [172]. This effect is at least partly mediated by TRPV1 blockage: it inhibited the calcium influx elicited by capsaicin in spinal cord synaptosomes with an IC50 of 44 nM and was able to displace [3*H*]-resiniferatoxin binding (IC50 = 47; 21–119 nM). Eriodictyol (4.5 mg/kg) induced antinociception in the intraplantar capsaicin test and reduced the thermal hyperalgesia and mechanical allodynia elicited by CFA paw injection. Although its interference with the proton binding mode of TRPV1 was not evaluated, a dosage of 4.5 mg/kg antagonizes the effect of capsaicin, but does not induce hyperthermia in mice [165].

Naringin, a main flavonoid of citrus fruits, is a second-generation antagonist of TRPV1 receptors. It inhibited capsaicin-stimulated TRPV1 activation in a concentration-dependent manner. It did not interfere with proton-stimulated activation. Molecular docking indicated that naringin has a different binding position than capsaicin. The most stable binding site between naringin and TRPV1 was predicted to be near the S1–S2 loop located at the extracellular side, with hydrogen bonds being established between the hydroxyl moieties on the 2-phenyl, on the oses and from the positions 5 and 7 of the chroman nucleus with Asn628, Asp471, Gly470 and Arg474, respectively. Mutagenesis studies indicated that residues Asp471 and Asn628 of TRPV1 were only involved in the binding to naringin but not capsaicin. This could also explain the lack of interference with proton activation, as the interaction position of protons is between S5 and S6 [168].

**Figure 5:**
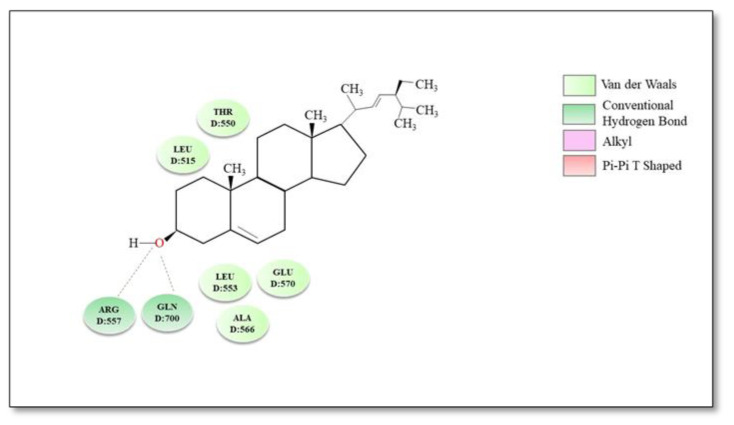
Molecular docking—2D diagram of stigmasterol with TRPV1 receptor.

Various sterol derivatives were reported to act as TRPV1 antagonists: α-spinasterol (0.3 µmol/kg p.o.) and stigmasterol (0.3 µmol/kg p.o.) counteracted capsaicin-induced nociception 1 h after treatment with 58 ± 4% inhibition and 40 ± 7% inhibition of pain, respectively [173]. However, α-spinasterol and stigmasterol did not induce adverse reactions such as alteration of locomotor activity [16] and did not significantly change Tb; therefore, we considered them second-generation antagonists. A molecular docking study indicated that stigmasterol establishes via the hydroxyl group in position C-3 of the steroid skeleton two hydrogen bonds with residues Arg557 and Gln700, respectively, while rings A and B are involved in multiple hydrophobic interactions with residues Thr550, Leu515, Leu553, Ala566 and Glu570 [174]. The best binding conformation and possible binding modes of stigmasterol and TRPV1 are illustrated in Figure 5.

Beta-sitosterol is a sterol widely found in the vegetal world. β-Sitosterol showed analgesic effect in tail flick and hot-plate tests [175]. It was also shown to inhibit capsaicin-induced Ca^2+^ influx. Molecular docking indicates it fits in the same pocket of TRPV1 receptors as capsazepine [176]. The major interactions between TRPV1/antagonists, as well as the involved amino acid residues and chemical moieties, are presented in Table 2.

## 4. Discussions

Ligands of TRPV1 possess a high structural variety. However, some common elements may be identified. Classical TRPV1 agonists, such as capsaicin and related compounds, possess common structural elements. They all possess three important pharmacophores: a polar head, body (ester, ketone, hydroxyketone and amide moiety) and a hydrophobic tail (usually an alkyl group) [177]. We identify as essential pharmacophore features in the linker region a hydrogen bond acceptor, a hydrogen bond donor and a ring feature, in conformity with previous reports [101,107,178]. If an adequate length of the tail is provided, the vanillyl moiety of these classical activators binds to residues Thr550 and Trp549, located in transmembrane regions 3 and 4 (S3/4) of rat and human TRPV1 via hydrogen bonds [134]. The alkaloid evodiamine also possesses these critical TRPV1 recognition elements: a hydrogen bond acceptor (formyl carbonyl oxygen) and a hydrogen bond donor (indole nitrogen) [107]. Furthermore, rings 1 and 5 within its structure act as two clusters for establishing hydrophobic interactions [107]. Similar to capsaicin, it occupies the binding pocket formed by Ser510, Tyr511, Leu515, Tyr555, Met568, Ile569, Glu570 and Lys571. However, owing to the lack of the polar head, it has a different spatial conformation. Thus, it establishes hydrogen bonds with other residues than vanilloid derivatives—Ile569 and Lys571 [107].

Piperine has a similar behavior: it also binds to the same ligand-binding pocket as capsaicin but in different conformations. Although piperine has an aromatic heterocyclic ring and a fatty chain similar to capsaicin, its structural variation leads to a different type of TRPV1 activation. Its head (di-oxol group) is further away from the carbonyl, which in capsaicin participates in another hydrogen bond, and it has no hydroxyl, thus it does not interact with residues Thr550 and Trp549. In piperine, the aliphatic tail is replaced by a piperidine ring. These structural changes lead to an interaction with Thr671, thus altering the pore-forming S6 segment, critical for channel opening [106]. Previous reports suggest that the lower part of S6 in TRPV1 opens similar to a gate in the presence of specific ligands. The point at which S6 bends in the TRPV1 structure is Tyr671. Tyr761 constitutes the most constricted point in the lower gate of the TRPV1 channel structure, and it might contribute to the allosteric coupling between thermal- and capsaicin-dependent activation mechanisms [179].

Information is lacking on the interactions established by unsaturated dialdehyde terpenes and TRPV1. However, we must highlight the presence of numerous oxygen atoms which can behave like hydrogen bond acceptors. Furthermore, sesquiterpenes with an α,ß-unsaturated 1,4-dialdehyde moiety have been shown to undergo Paal–Knorr condensation reactions with lysine residues of TRP channels [180].

Monoterpenes which lack the vanilloid head and the aliphatic tail target different binding sites and have a hydrocarbon backbone that establishes various hydrophobic interactions with the outer pore region. They are not associated with the pore dilated open state, which is associated with capsaicin-induced currents and some of them, such as eugenol, are mode-selective. The mode-specific antagonistic properties of eugenol can be exploited to develop a lead compound for pain treatment without adverse effects (129).

**Figure 6:**
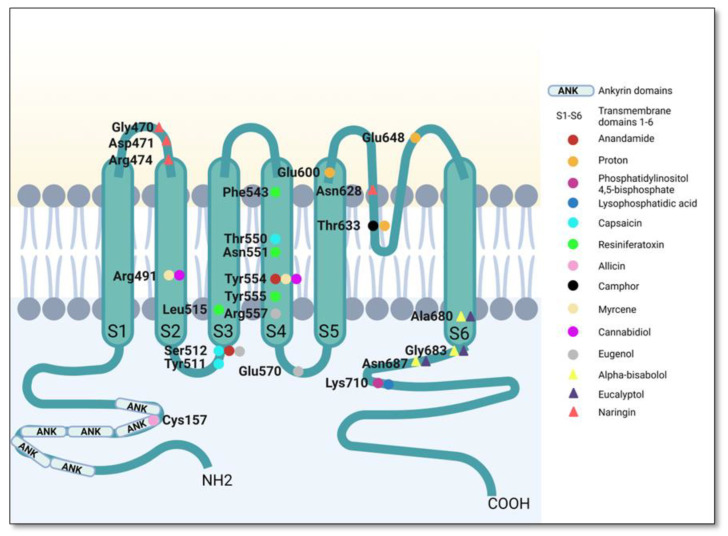
Main interactions of TRPV1 modulators.

From the antagonist class, we highlight the chemical class of sterols, with various members from this class behaving similarly in terms of not interfering with the Tb. Their unifying structural feature is the presence of a hydroxyl group flanked by the tetracyclic ring, which establishes multiple hydrophobic interactions that stabilize the complex target/ligand. Representative agonists and antagonists and the principal interaction sites are illustrated in Figure 6.

In conclusion, understanding the complex interactions between a complex molecular target such as TRPV1 and its various ligands would allow us to effectively modulate the former. We identified monoterpenes and sterols as promising scaffolds for developing TRPV1 modulators lacking hyperthermic effect.

## Figures and Tables

**Table 1:** Main interactions of TRPV1 agonists.

Plants	Active Compounds	Chemical Class	Key Structures	TRPV1 Residues	Type of Bond	Reference
*Capsicum* spp.	Capsaicin	Vanilloids	Amide oxygen Aromatic hydroxyl	Tyr551 Glu 571	Hydrogen bond	[134,135]
			Vanillyl moiety	Leu515, Ile573, Phe587, Leu669 and Thr550	Hydrophobic interactions	
			Aliphatic tail	Met547, Ala665, Phe543 and Tyr511		
*Euphorbia resinifera*	Resiniferatoxin		Vanillyl moiety	Tyr555	Hydrophobic interactions	[94,134]
			Side chains	Leu515, Phe543 and Asn551	Hydrophobic interactions	
*Cannabis sativa*	Cannabidiol	Phytocannabinoids	Diverse C within the structure	Tyr554 and Arg491	Hydrophobic interactions	[129]
*Thymus vulgaris* *Cannabis sativa*	Myrcene	Monoterpenes	Dimethyl group	Arg491, Tyr555, Phe434 and Asn437	Hydrophobic interactions	
			Diverse C within the structure	Ser512, Tyr554, Phe488, Phe516 and Glu513		
*Cinnamomum camphora*	Camphor		Diverse C within the structure	Trp427, Lys432, Phe496, Phe490, Arg491, Tyr487, Leu638, Phe639 and Leu648	Hydrophobic interactions	[125]
*Piper nigrum; Piper longum; Piper officinarum*	Piperine	Alkaloids	Carbonyl group	Thr551	Hydrogen bonds	[106]
*Evodia rutaecarpa*	Evodiamine		Ring 1 (the indole’s benzene ring)	Tyr511 (human) and Tyr514 (rabbit)	Hydrophobic interactions	[107]
			Ring 5 (the benzene of the quinazoline moiety)	Tyr555 (human) and Tyr558 (rabbit)	Hydrophobic and aromatic π–π interactions	
			Indole nitrogen	Ile569 (human) and Ile572 (rabbit)	Hydrogen bond (donor)	
			Formyl carbonyl oxygen	Lys571 (human) and Lys574 (rabbit)	Hydrogen bond (acceptor)	
*Allium sativum*	Allicin	Thiosulfinates	Thiosulfinate group	Tyr550 and Tyr554	Hydrogen bond	[136]
				Glu510	Attractive charge	
			Diverse C within the structure	Ile569, Ala566, Arg557, Leu553, Ser512, Asn551 and Leu515	Van der Waals	
*Eugenia caryophyllata; Ocimum gratissimum; Cinnamomum zeylanicum*	Eugenol	Allylbenzenes	Hydroxyl group on vanillyl moiety	Glu570, Arg557 and Ser512	Hydrogen bond	[137]
			Allyl chain	Leu553, Ala566, Ile569 and Ile573	Hydrophobic interactions	
*Zingiber officinalis*	6-gingerol	Guaiacols	Carbon side chain	Phe49 and Phe93	Hydrogen bond	[138]
			Carbonyl group	Ile293		
			Hydroxyl group of aliphatic chain	Lys285		
	8-gingerol		Diverse C within the side chain	Phe49, Phe58, Phe93 and		
			Carbonyl group	Ile293		
			Hydroxyl group of aliphatic chain	Lys285		
	10-gingerol		Diverse C within the side chain	Phe49, Phe58, Phe93 and Gly210		
			Carbonyl group	Ile293		
			Hydroxyl group of aliphatic chain	Lys285		
	6-shogaol	Phenols	Diverse C within the side chain	Phe49, Phe93		
			Carbonyl group	Ile293		
	8-shogaol		Diverse C within the side chain	Phe49, Phe58 and Phe93		
			Carbonyl group	Ile293		
	10-shogaol		Diverse C within the side chain	Phe49, Phe58 and Phe93		
			Carbonyl group	Ile293		
	Zingerone		Ketone group	Thr551	Hydrogen bond	[98]
			Vanillyl group	Glu571 and Thr671		
	6-shogaol and 6-gingerol	Phenols and guaiacols	Ketone group	Thr551	Hydrogen bond	
			Vanillyl group	Glu571		
	2-paradol, 4-paradol and 8-paradol	Phenols	Hydroxyl group	Leu32 and Thr28	Hydrogen bond	[97]
	6-paradol			Glu140, Gln135 and Gln143		

**Table 2:** Main interactions of TRPV1 antagonists.

Influence on Body Temperature	Plants	Active Compounds	Chemical Class	Key Structures	Residues TRPV1	Observation	Reference
Unknown	*Matricaria recutita* and *Myoporum crassifolium*	Alpha-bisabolol	Sesquiterpenoid	Diverse C within the structure	Ala680 and Asn687	Hydrophobic interactions	[158]
				Hydroxyl group in position C-2	Gly683	Hydrogen bond	
Does not interfere	*Vernonia tweedieana*	Stigmasterol	Sterols	Hydroxyl group in position C-3 of the steroid skeleton	Arg557 and Gln700	Hydrogen bond	[174]
				Rings A and B of the steroid skeleton	Thr550, Leu515, Leu553, Ala566 and Glu570	Hydrophobic interactions	
	*Arbutus andrachne*	Beta-sitosterol	Sterols	Hydroxyl group in position C-3	Phe543	Hydrogen bond	[176]
				Sterolic nucleus	Leu515, Ala546, Met547, Leu553, Ala665 and Leu669	Hydrophobic interactions	
	Citrus peel	Naringin	Flavonoids and corresponding glycosides	Hydroxyl group on the phenyl moiety	Asn628	Hydrogen bond	[168]
				Hydroxyl groups on the oses	Asp471 and Gly470		
				Hydroxyl groups in position C-5, C-7	Arg474		
				Phenyl group in position C-2	Val457	Hydrophobic interactions	
	*Eucalyptus globulus*	Eucalyptol	Monoterpenoids	Cineol ring	Ala680, Gly683 and Asn 687	Hydrophobic interactions	[163]

## Data Availability

Not applicable.

## Abbreviations

AEA, anandamide; ATP, adenosine triphosphate; CFA, complete Freund’s adjuvant; CHO, Chinese hamster ovary; DRG, dorsal root ganglion; EC50, half-maximal effective concentration; GEPR-3s, genetically epilepsy-prone rats; HaCaT, human epidermal keratinocyte line; HEK293, human embryonic kidney 293; hTRPV1, human transient receptor vanilloid 1; IC50, half-maximal inhibitory concentration; mTRPV1, mouse transient receptor vanilloid 1; NADA, N-arachidonoyl dopamine; OEA, N-oleoylethanolamine; OLDA, oleoyldopamine; PIP2, phosphatidylinositol 4,5-bisphosphate; PME, pinosylvin methyl ether; rTRPV1, rat transient receptor vanilloid 1; RTX, resiniferatoxin; Tb, body temperature; TRPA, transient receptor potential ankyrin; TRPV1, transient receptor vanilloid 1; S1–S6, transmembrane domains 1–6; VBP, vanilloid binding pocket.
